# The experience of Hamad General Hospital collaborative anticoagulation clinic in Qatar during the COVID-19 pandemic

**DOI:** 10.1007/s11239-020-02276-4

**Published:** 2020-10-04

**Authors:** Ibtihal Abdallah, Asma Eltahir, Liam Fernyhough, Ahmed El-Bardissy, Rana Ahmed, Mohammed Abdulgelil, Dina Elgaily, AbdulMoqeeth Mohammed, Ameena Jassim, Loluwa Barakat, Mazen Al-Ansari, Mehak Javed, Raja Alkhawaja, Abdel-nasser Elzouki

**Affiliations:** 1 Clinical Pharmacy Department, Hamad General Hospital, Hamad Medical Corporation, Doha, Qatar; 2 Hematology Department, National Center for Cancer Care and Research, Hamad Medical Corporation, Doha, Qatar; 3 Weill Cornell Medicine, Doha, Qatar; 4 Internal Medicine, Hamad General Hospital, Hamad Medical Corporation, Doha, Qatar

**Keywords:** Anticoagulation, Clinical pharmacy, Collaborative practice, COVID-19, Social distancing, Warfarin

## Abstract

Hamad General Hospital Anticoagulation Clinic is one of the largest collaborative-practice clinics of its type in Qatar. The patients being followed at this clinic are typically complex and vulnerable. During the coronavirus disease 2019 pandemic, measures were implemented at the clinic to minimize the exposure of patients and healthcare providers to the acute respiratory syndrome coronavirus-2 and to promote social distancing. These measures included extending INR-recall period, transitioning to direct oral anticoagulant drugs whenever feasible, home visits to elderly and immunocompromised patients for INR testing, establishing an anticoagulation hotline, and relocation of warfarin dispensing from the main pharmacy to the anticoagulation clinic. In addition, the clinic shifted its multidisciplinary team meetings onto an online platform using Microsoft Teams. Telehealth consultations were extensively utilized to closely follow up with the patients and ensure that anticoagulation efficacy and safety remained optimal. The aim of this paper is to share our experience and describe the measures adopted by the clinic as part of the Hamad Medical Corporation response to the emerging situation.

**Electronic supplementary material:**

The online version of this article (10.1007/s11239-020-02276-4) contains supplementary material, which is available to authorized users.

## Highlights

- Anticoagulation drugs’ management is complex.
- Pharmacist-led collaborative anticoagulation clinic is a well-recognized model of care.
- COVID-19 pandemic forced the reshape of clinical practice.
- We employed multiple measures to care for our patients during the pandemic.
- Telemedicine is an important approach to help social distancing.

## Introduction

In late 2019, an unusual type of pneumonia was identified in the city of Wuhan, in the Hubei Province of China. Later, the severe acute respiratory syndrome coronavirus 2 (SARS-CoV-2) virus was found to be the causative organism of this pneumonia, and the disease was given the name coronavirus disease 2019 (COVID-19) in February 2020 by the World Health Organization. COVID-19 rapidly became a pandemic, infecting over 10 million individuals at the date this article is written, and causing the death of over half a million individuals around the world [1, 2].

Anticoagulant drugs are agents that block the coagulation factors thereby reduce the risk of blood clotting. They are used in various clinical conditions, such as atrial fibrillation and venous thromboembolism treatment and prophylaxis. They have been classified as high-risk medications by the Institute of Safe Medication Practices (ISMP) because of the high potential for adverse outcomes associated with their use.^3^ Problems include major and minor bleeding, drug interactions with several drug classes, and a high risk of thromboembolism when anticoagulants are interrupted [3].

Due to the complexity of anticoagulation drugs’ management, pharmacist-led collaborative anticoagulation clinics within the setting of family or general medicine clinics have been advocated. Such approaches are patient-centered and have shown favorable clinical outcomes in terms of efficacy and safety of anticoagulation [4, 5].

The COVID-19 pandemic has forced health systems to quickly adapt to telehealth for a large portion of patient care services so as to minimize patients’ and healthcare workers’ risk of exposure. With regard to the management of anticoagulant drugs, the Anticoagulation Forum (acforum.org) has endorsed two approaches: 1. *to extend the INR recall interval*, and 2*. to switch from warfarin to Direct Oral Anticoagulants (DOACs) wherever possible without compromising patient safety* [6, 7].

## Collaborative practice at Hamad General Hospital anticoagulation clinic

Hamad General Hospital (HGH) anticoagulation clinic (ACC) replaced the traditional warfarin clinic and embraced the physician-pharmacist collaborative practice starting in 2016. The ACC clinic functions under the umbrella of the internal medicine ambulatory care service. Its team consists of clinical pharmacists working collaboratively with a team of physicians, and it is supported by nursing and patient care assistants.

When a new patient is referred to the clinic, the first assessment is done and documented by one of the clinic physicians. Subsequent follow up visits are led by the clinical pharmacist running the clinic. After the clinic nurse checks the INR using the CoaguChek® XS Plus point-of-care (POC) device, the patient is interviewed by the clinical pharmacist. Decisions on dose modification, follow up visits, and further investigations (such as complete blood count, renal function test and in-lab INR, etc.) are decided upon and ordered by the clinical pharmacist. All necessary documentation is done by the clinical pharmacist for these follow up visits. In certain scenarios, such as a high INR (> 5) or patient complaints that require physical examination, the patient is referred to the clinic physicians who are available at all times for support. (please refer to Appendix 1 for HGH-ACC collaborative agreement). Patients who are being managed using DOACs are also referred to this clinic for follow up and monitoring of their therapy effectiveness, safety, compliance, and regular laboratory parameter checks.

HGH-ACC is a high-volume service. In 2019, around 450 clinics were run in which over 5000 patients’ visits took place, an average of 20 patients per day. The average INR recall interval was 23 (± 14) days and the mean Time in Therapeutic Range (TTR) was 62.6%. Although the TTR could be higher, the calculation is affected by how long the patients have been on warfarin and is seen to be lower studies where the first month of warfarin is included (TTR approximately 56%), and higher in studies that exclude patients in the first three months of warfarin treatment (TTR approximately 75%) [8]. As we do include patients in their initial months of warfarin therapy in our TTR calculation, our TTR of 62.6% compares favourably. The TTR at our center is also likely an underestimate as we did not exclude from the calculation patients who had their warfarin withheld purposefully for procedures or bleeding.

During the COVID-19 pandemic, physical distancing measures were required and the number of patients visiting the clinic had to be reduced to avoid crowding of patients and minimize the risk of acquiring SARS-CoV2 infection. The HGH-ACC team put together a multi-faceted program to limit the number of patients physically attending the clinic by building upon the two main approaches endorsed by the Anticoagulation Forum and incorporating other novel and innovative strategies. The components of this program are described here.

## Advanced chart review

The HGH-ACC team reviewed the daily list of clinic appointments 2-3 days prior to the given date. The patients’ electronic files on Cerner® (Cerner Corporation, Kansas City, Missouri, U.S.A) were meticulously examined to determine whether a given patient was eligible for extending the INR recall period, transitioning to DOACs, or needed to attend their clinic visit as planned. The patients were contacted accordingly. All calls and plans were documented in the patients’ files.

## Extension of INR recall period

The recall period for INR follow up depends on the INR stability. Recall visits are initially at short intervals, but they are then extended based on the achievement of consecutive therapeutic range INR readings. The American College of Chest Physicians (ACCP) Antithrombotic Guidelines (2012) recommend that the follow up interval can be as long as 12 weeks for stable patients with multiple INR readings within the therapeutic range and a low risk of bleeding [9]. This recommendation was endorsed by the Anticoagulation Forum in response to the COVID-19 situation [6]. As per the Michigan Anticoagulation Quality Improvement Initiative (MAQI), the follow up period depends on the number of therapeutic INR readings. For example, if the patient has one in-range INR, the follow up visit should take place within a week, two-weeks follow up in case of two therapeutic INR readings, and so on, up to a maximum of 12 weeks follow up interval [6, 10].

**Fig. 1:**
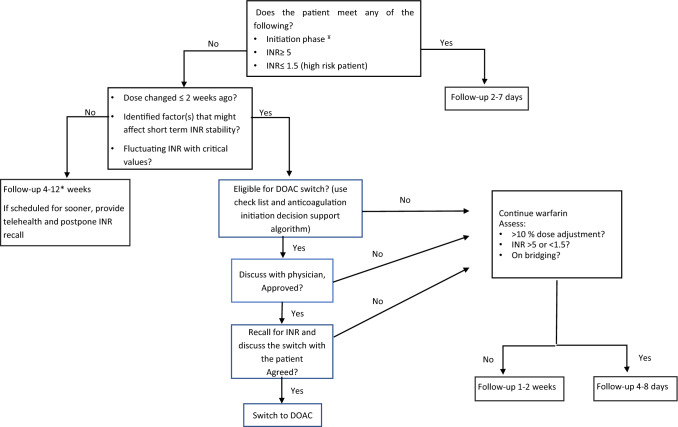
Warfarin management decision algorithm. ¥ follow the standard initiation nomogram. *Decision on INR recall interval base on the duration in therapeutic INR: 4 weeks recall after 4 weeks of therapeutic INR, up to a of maximum of 8 weeks. 12 weeks interval should be provided only if patient is maintained on the same dose for the previous 6 months and TTR ≥ 65%. Apply clinical judgment to decide upon recall interval between 8–12 weeks

To help us stratify our patients (those who needed to attend the clinic as scheduled versus those who could have their appointments rescheduled), we reviewed the recommendations from multiple respected anticoagulation institutes; which we summarized into a decision-making support algorithm to guide and facilitate the process (Fig. 1).

## Using telehealth consultations

Using this evidence-based approach, we were able to significantly reduce the number of patients physically attending the clinic. However, telehealth was provided for all patients whose INR recall visits were extended to ensure that the safety of patients was not compromised. Telehealth was used to ensure that each patient was taking the correct dose, maintaining a consistent diet, and to assess the commencement or cessation of medications that might affect the INR. Telehealth was also provided for all patients on DOACs. Nevertheless, adherence to warfarin and DOACs was proactively addressed in the telehealth communication with each patient.

## Transitioning from warfarin to DOACs

As patients who take DOACs do not need repeated measurement of the INR, they are particularly suited to follow up using telehealth. Each patient was therefore reviewed for their suitability to transition from using warfarin to using a DOAC for anticoagulation.

**Fig. 2:**
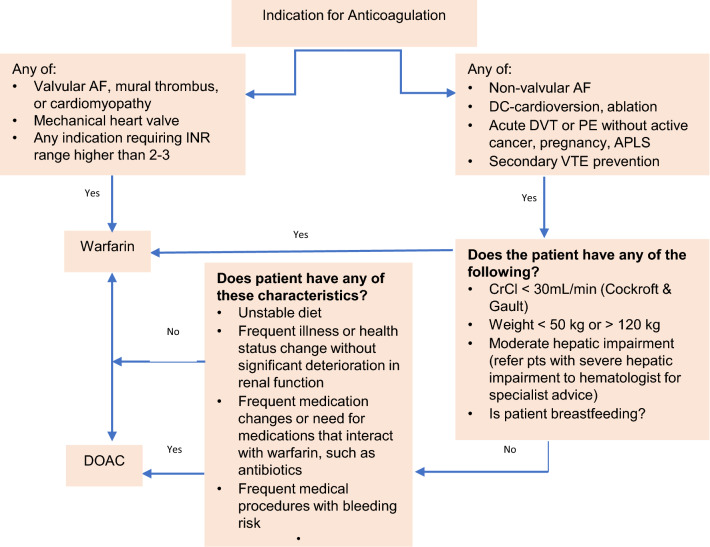
Anticoagulation initiation decision-support algorithm. *AF* atrial fibrillation, *DC* direct current, *DVT* deep vein thrombosis, *PE* pulmonary embolism, *APLS* antiphospholipid antibody syndrome, *VTE* venous thromboembolism, *CrCl* creatinine clearance, *DOAC* direct oral anticoagulant

We designed a decision-support tool (Table 1 and Fig. 2) to comprehensively cover contraindications and supporting factors for the transition. Our main aim was to transition the patients who had a labile INR and no contra-indications to DOACs after patient counselling.

## Home INR testing for elderly and immunocompromised patients

A large-scale collaborative project between the anticoagulation clinical pharmacists, physicians, and Home Health Care Service (HHCS) was initiated. The aim of this project was to facilitate the INR monitoring for the fragile, high-risk, and immunocompromised patients who were not eligible for extending the INR recall period nor transitioning to DOACs. These patients were first identified by our team of clinicians. The inclusion criteria were adapted from the *Centers for Disease Control and Prevention (CDC)* classified high risk patients and summarized into Table 2 [11]. The list of patients was provided to the HHCS coordinator. The coordinator reviewed the list to ensure that patients met the project inclusion criteria, and then contacted eligible patients to arrange for the visits. The patients were visited at the comfort of their homes by the HHCS team of nurses for INR testing. The INR value was then recorded into the patient electronic medical record on Cerner® by the HHCS nurse. A clinical pharmacist then provided comprehensive telehealth, inquiring about the current dose of warfarin, ensuring that no signs or symptoms of bleeding or thrombosis were present, providing education, and attending to any patient concerns.

**Table 1:**
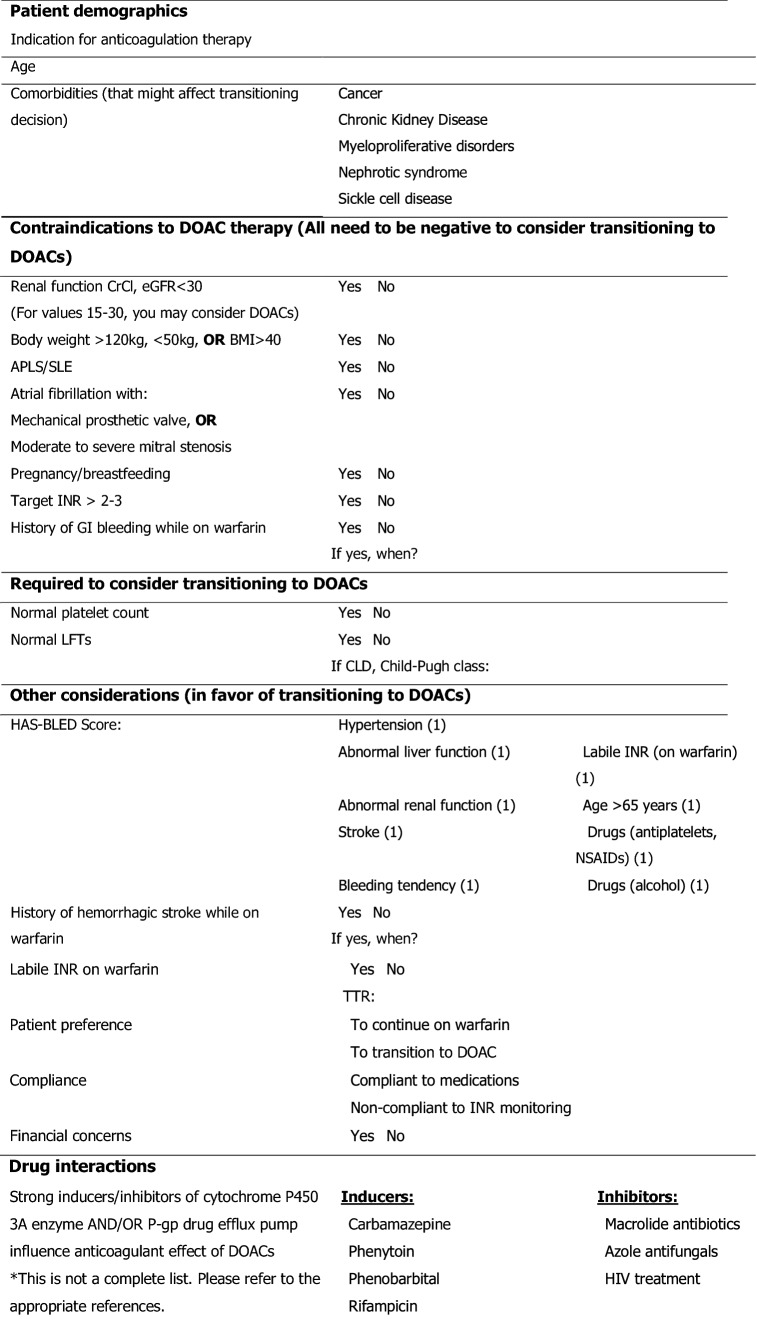
Transitioning from warfarin to DOACs checklist

**Table 2:** High risk patient groups

High-risk patients
People older than 65 years old
People of all ages with any of the following conditions:
Cancer
Bone marrow transplant
Solid organ transplant
Stem cells for cancer treatment
Genetic immune deficiencies
Human Immunodeficiency Virus (HIV) infection/acquired immunodeficiency syndrome (AIDS)
Use of oral or intravenous corticosteroids or other immunosuppressants infections (e.g., mycophenolate, sirolimus, cyclosporine, tacrolimus, etanercept, rituximab)
Known respiratory illnesses, e.g. asthma, chronic obstructive pulmonary disease (COPD)

This project significantly reduced the number of elderly or immunocompromised patients physically attending the clinic, and hence reduced their chances of exposure to SARS-CoV-2.

## Establishing an Anticoagulation Hotline

The Qatar System Wide Incident Command Committee (SWICC) has established a national Coronavirus Disease Call Center, which received patients’ calls and had designated healthcare providers available around the clock to attend to all healthcare inquiries, including anticoagulation.

In addition, the HGH-ACC team took a further measure to ensure that patients had easy and quick access to anticoagulation services in case of concerns or anticoagulation emergencies. A corporate mobile phone number was designated to the clinic as an anticoagulation hotline, and the patients were provided with this number to call or text at any time. Examples of issues managed by this line include responding to INR results released after the clinic hours, emphasizing plans by sending text messages, and dose clarification. This reduced the patients’ need to walk-in to clinic for simple inquiries and hence reduced unnecessary patient and healthcare worker exposure.

## Relocation of Warfarin Dispensing to the Anticoagulation Clinic

A nation-wide project was led by the Pharmacy Department across Hamad Medical Corporation to verify and dispense medications utilizing telehealth and the national post courier in Qatar, Qatar Post (QPost). Once prescribed, the pharmacy dispenses and posts the medications directly to the patient doorstep.

To avoid any unexpected delay, and to minimize additional waiting time and person-to-person exposure of our patients, warfarin was dispensed at the clinic at the end of each encounter instead of the usual dispensing process at the hospital pharmacy. This project was coordinated and supported by Hamad General Hospital Pharmacy Department to contribute to patient safety and reduce the exposure during the pandemic.

## Multidisciplinary Team (MDT) Meetings

Prior to COVID-19, the clinic team met monthly in an MDT meeting that included the ACC clinical pharmacists and physicians, internal medicine consultants, and hematology consultants. The aim of the MDT meetings was to discuss and reach consensus regarding the management of complicated patient issues. Examples of issues classically discussed in the MDT meetings were the eligibility of certain complex patients for transitioning from warfarin to DOACs and duration of anticoagulation therapy when a straightforward answer was not available in the medical literature.

COVID-19 caused an interruption in these meetings. However, as the clinic workflow became clear and smooth, the team resumed virtual MDT meetings through Microsoft Teams® (Microsoft Corporation, Redmond, Washington, United States).

## Conclusion

COVID-19 caused significant disruption to normal healthcare services and gave rise to the need for innovative solutions that would maintain standards of patient care despite physical distancing. The multifaceted systems approach that has been described here may well function as a model for health care during COVID-19 elsewhere and result in long lasting changes, even after the COVID-19 pandemic has subsided. Future research will evaluate the efficacy and the safety of these approaches, and their implications for the clinical outcomes of this patient population.

## Electronic supplementary material

Below is the link to the electronic supplementary material.

Supplementary file1 (DOCX 73 kb)
