# Supplementary material for: The experience of Hamad General Hospital collaborative anticoagulation clinic in Qatar during the COVID-19 pandemic
Source: J Thromb Thrombolysis. 2020 Oct 4;52(1):308–14. doi: 10.1007/s11239-020-02276-4 (PMC7533116; doi:10.1007/s11239-020-02276-4)
Supplement: Supplementary file 1 — Supplementary file1 (DOCX 73 kb) [file 11239_2020_2276_MOESM1_ESM.docx]

Role of the Anticoagulation clinic clinical pharmacist:

Clinical pharmacists* will be responsible for management of anticoagulation therapy at the clinic by providing structured and continuous patient education, screening, resolving and reporting drug­drug interactions, drug-food interactions, and non-adherence. The clinical pharmacists will monitor anticoagulation therapy, make dose adjustments and recommendations for safer use of alternative drugs that may not interact with warfarin/DOACs. The clinical pharmacist is responsible for documentation of anticoagulation visits.

***The clinical pharmacist will alert a physician attending the anticoagulation clinic if the patient meets any of the following criteria:***

1. The patient is complaining of any signs/symptoms that may be consistent with an arterial or venous thrombotic event.
2. Supratherapeutic INR value associated with bleeding or an INR value ≥ 5.0
3. Any signs of occult or active bleeding.
4. Patient who has repeated problems with noncompliance to medications, blood tests or clinic visits.

The clinical pharmacists will utilize Hamad Medical Corporation (HMC) approved clinical guidelines for this service. The clinical pharmacist will also seek physician input when needed.

***The clinical pharmacist*** will be responsible for renewal and dose adjustment and switch** of anticoagulant medications (co-signed by the physician attending the anticoagulation clinic)

These medications include:

- Warfarin
- Dabigatran
- Rivaroxaban
- Apixaban
- Enoxaparin
- Dalteparin

***Documentation: The anticoagulation clinic clinical pharmacist*** will document the patient visit and therapy plan in the warfarin therapy plan in Cerner Power chart.

*who had certification in the anticoagulation management for pharmacists, and passed the training period in the anticoagulation clinic.

**Switch between oral anticoagulants (warfarin to DOAC or vice versa will be done after physician approval)

NO

***Indications for referral to the physician attending the anticoagulation clinic:**

- Any patient found to be experiencing worsening symptoms, new symptoms, or new physical findings suggesting bleeding or thromboembolic event.
- INR > 5 with or without bleeding.
- Evaluation of the need for the anticoagulation or to revise therapy plan.
- Non-compliance
- INR out of the therapeutic range for 3 consecutive visits
- New patients presenting to Anticoagulation clinic
- Patient needing change of anticoagulation (Warfarin to DOACs or vice versa)

INR within the therapeutic range

INR out of the therapeutic range

The anticoagulation clinic clinical pharmacist will follow the protocol and adjust the dose accordingly. Give the appropriate education, and schedule the follow up visit

The anticoagulation clinic clinical pharmacist will instruct the patient to continue the same dose regimen, educate and schedule the follow-up visit

Document the patient visit in the warfarin therapy chart and order the medication in the power chart

Medication order is to be cosigned by the anticoagulation clinic MD

Check out

New patient

Follow-up patient

To be seen by the anti-coagulation clinical pharmacist, review the INR and inspect for any anticoagulation related problems

To be seen by the MD

Document the visit in the warfarin therapy chart, order the medication and schedule follow up visit in the power chart

Patient presenting to the anticoagulation clinic if on direct oral anticoagulant will skip the INR monitoring and be stratified immediately to new or follow-up patient

Indication to refer the patient to the anticoagulation clinic MD*

Yes

If patient is on vitamin K antagonist, the nurse will measure the INR by point-of-care device and document the result in Cerner, and then stratify the patients to either new or follow-up patient

**Addendum 1: Anticoagulation clinic flow diagram**

For any patient presenting to the anticoagulation clinic for follow-up, the anticoagulation clinical pharmacist should check for the end of therapy date and review the indication for the anticoagulation

The patient is at the end of therapy or Eligible to discontinue anticoagulation

NO

Yes

Clear Discontinuation plan, and no post discontinuation follow-up plan (i.e. provoked VTE without sequel)

No documented end of therapy plan, or there is post-discontinuation follow-up plan (e.g. Thrombophilia, imaging etc.)

Anticoagulation clinic clinical pharmacist shall discharge the patient, educate the patient and document the visit in the warfarin therapy form

Discharge

Refer to the physician attending the anticoagulation clinic

Follow the regular follow-up workflow

**Addendum 2: Anticoagulation clinic discharge and discontinuation of therapy flow diagram**
